# Improving chronic disease prevention and screening in primary care: results of the BETTER pragmatic cluster randomized controlled trial

**DOI:** 10.1186/1471-2296-14-175

**Published:** 2013-11-20

**Authors:** Eva Grunfeld, Donna Manca, Rahim Moineddin, Kevin E Thorpe, Jeffrey S Hoch, Denise Campbell-Scherer, Christopher Meaney, Jess Rogers, Jaclyn Beca, Paul Krueger, Muhammad Mamdani

**Affiliations:** 1 Department of Family Community Medicine, University of Toronto, 500 University Avenue, Toronto, Ontario M5G 1V7, Canada; 2 Ontario Institute for Cancer Research, Toronto, Ontario, Canada; 3 Department of Family Medicine, University of Alberta, 901 College Plaza, Edmonton, Alberta T6G 2C8, Canada; 4 Applied Health Research Centre, Li Ka Shing Knowledge Institute, St. Michael’s Hospital, 193 Yonge Street, Toronto, Ontario M5B 1M8, Canada; 5 Dalla Lana School of Public Health, University of Toronto, Toronto, Canada; 6 Centre for Excellence in Economic Analysis Research, The Keenan Research Centre, Li Ka Shing Knowledge Institute, St. Michael’s Hospital, 30 Bond Street, Toronto M5B 1W8, Canada; 7 Pharmacoeconomics Research Unit, Cancer Care Ontario, Toronto, Canada; 8 Centre for Effective Practice, 203 College Street, Suite 402, Toronto M5T 1P9, Canada; 9 Institute for Health Policy, Management and Evaluation, Faculty of Medicine, University of Toronto, Toronto, Canada; 10 Leslie Dan Faculty of Pharmacy, University of Toronto, Toronto, Canada

**Keywords:** Primary care, Family practice, Pragmatic trial, Chronic disease prevention, Cancer screening, Facilitation

## Abstract

**Background:**

Primary care provides most of the evidence-based chronic disease prevention and screening services offered by the healthcare system. However, there remains a gap between recommended preventive services and actual practice. This trial (the BETTER Trial) aimed to improve preventive care of heart disease, diabetes, colorectal, breast and cervical cancers, and relevant lifestyle factors through a practice facilitation intervention set in primary care.

**Methods:**

Pragmatic two-way factorial cluster RCT with Primary Care Physicians’ practices as the unit of allocation and individual patients as the unit of analysis. The setting was urban Primary Care Team practices in two Canadian provinces. Eight Primary Care Team practices were randomly assigned to receive the practice-level intervention or wait-list control; 4 physicians in each team (32 physicians) were randomly assigned to receive the patient-level intervention or wait-list control. Patients randomly selected from physicians’ rosters were stratified into two groups: 1) general and 2) moderate mental illness. The interventions involved a multifaceted, evidence-based, tailored practice-level intervention with a Practice Facilitator, and a patient-level intervention involving a one-hour visit with a Prevention Practitioner where patients received a tailored ‘prevention prescription’. The primary outcome was a composite Summary Quality Index of 28 evidence-based chronic disease prevention and screening actions with pre-defined targets, expressed as the ratio of eligible actions at baseline that were met at follow-up. A cost-effectiveness analysis was conducted.

**Results:**

789 of 1,260 (63%) eligible patients participated. On average, patients were eligible for 8.96 (SD 3.2) actions at baseline. In the adjusted analysis, control patients met 23.1% (95% CI: 19.2% to 27.1%) of target actions, compared to 28.5% (95% CI: 20.9% to 36.0%) receiving the practice-level intervention, 55.6% (95% CI: 49.0% to 62.1%) receiving the patient-level intervention, and 58.9% (95% CI: 54.7% to 63.1%) receiving both practice- and patient-level interventions (patient-level intervention versus control, P < 0.001). The benefit of the patient-level intervention was seen in both strata. The extra cost of the intervention was $26.43CAN (95% CI: $16 to $44) per additional action met.

**Conclusions:**

A Prevention Practitioner can improve the implementation of clinically important prevention and screening for chronic diseases in a cost-effective manner.

## Background

Most industrialized countries are facing an unprecedented rise in chronic disease, with many patients suffering from multiple chronic conditions [1,2]. Primary prevention and screening for chronic diseases are considered the best hope to curtail this rise [3,4]. Agencies such as the National Institute for Health and Clinical Excellence [5], Canadian Task Force on Preventive Health Care; [6] and the US Preventive Services Task Force [7] synthesize and grade their recommended chronic disease prevention and screening (CDPS) actions based on high level clinical trial evidence that these actions will lead to improved clinical outcomes. For example, it is estimated that 25% of all direct medical costs are attributable to a small number of excess risk factors such as smoking, obesity, physical inactivity and poor nutrition [8]. Even a 10% reduction in the prevalence of physical inactivity, for example, could lead to substantial reductions in direct health-care expenditures [2]. Similarly, improving screening rates can reduce colorectal cancer deaths by 15% to 33% [9] and cardiovascular risk assessment with blood pressure readings has been shown to reduce population level cardiovascular morbidity [10]. The challenge for healthcare systems worldwide is to improve rates of CDPS that are recommended based on high level evidence that they lead to improved outcomes [7].

Primary care, as the site of the patient’s medical home, [11] is an effective and efficient setting to provide evidence-based care [12]. Many of the evidence-based actions and strategies for CDPS are set in primary care and supported by clinical practice guidelines [13]. However, there remains a gap between recommended CDPS and actual practice, [14] due in part to the competing care demands on Primary Care Physicians. For example, one study found the time required to provide all recommended preventive services to a typical family practice is 7.4 hours per working day, which would leave little time for other patient care activities [14]. Furthermore, patients with mental illness are a vulnerable subgroup with high prevalence in primary care [15]. Due to the complexity of dealing with multiple medical issues, these patients experience a large gap in preventive care, making this an important subgroup to target for improved CDPS [16-18].

Evidence on how best to implement effective care has shown that tailored, active, and multifaceted interventions using an amalgam of strategies are most effective [19-22]. Among the effective strategies is practice facilitation, a process by which a trained individual – usually external to the practice and not involved in direct patient care [23] - supports primary care practices to improve the quality of care [24,25]. However, a modification of practice facilitation involving direct patient contact and applied to improving chronic disease prevention and screening concurrently for several chronic diseases has not previously been tested. We studied a multi-level [26] strategy using a modification of practice facilitation to implement *integrated* prevention and screening of chronic diseases (i.e., one that integrates heart disease, diabetes, cancer and the lifestyle factors associated with these diseases) based in primary care.

The objective of the trial “Building on Existing Tools to Improve Chronic Disease Prevention and Screening in Primary Care (the BETTER trial) was to improve uptake of clinically effective CDPS actions for primary prevention of heart disease and diabetes, screening for colorectal, breast and cervical cancers, and relevant lifestyle factors through an implementation trial of a multifaceted multi-level tailored intervention set in primary care and predicated on a model of practice facilitation compared to standard care.

## Methods

### Primary care team practices

The setting was urban Primary Care Team practices in two Canadian provinces identified by purposive sampling to obtain a mix of academic teaching practices and community non-teaching practices in different locations. Primary Care Team practices are integrated primary care delivery models, [27] that serve as their patients’ medical home [11]. These are general practices that provide first contact with the health care system including long-term person and family focused care for all of their patients’ health needs, including access to other resources such as specialty services. All participating practices had been using an electronic medical record (EMR) system to manage their practice for at least two years.

### Patients

Eligible patients were identified through the EMR and included all active patients (seen within the previous three years) age 40 to 65 years and rostered to one of the participating physicians. This age group was selected because of the applicability of most CDPS actions to this age group for both men and women. Patients were excluded if they were not able to give informed consent or attend the practice for the intervention. Patients were stratified into two mutually exclusive groups: Stratum 1: general medical patients and Stratum 2: patients with moderate mental illness (defined as a diagnosis of depression, anxiety or psychosomatic disorder within the previous 12 months plus two instances of diagnosis or one diagnosis and one prescription). All participants gave written informed consent.

### Design and intervention

**Figure 1:**
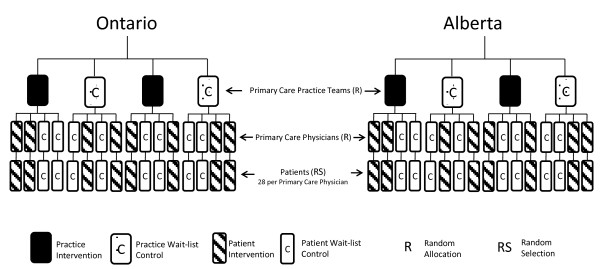
Trial design schema.

We conducted a pragmatic two-way factorial cluster randomized controlled trial (RCT) with physicians’ practices as the unit of allocation and individual patients as the unit of analysis involving a practice-level and patient-level facilitation intervention. The study was conducted in eight Primary Care Team practices, with four physicians in each team agreeing to participate for a total of 32 participating physicians. The eight Primary Care Team practices were randomly assigned to the practice-level intervention or wait-list control group. The four participating physicians within each Primary Care Team practice were randomly assigned to the patient-level intervention or wait-list control group (Figure 1). In this way participants were assigned to one of four groups: 1) both the practice-level and patient-level control; 2) both the practice-level and patient-level intervention; 3) the practice-level control and patient-level intervention; or 4) the practice-level intervention and patient-level control. Randomization was at the level of the physician (rather than the individual patient) to avoid contamination. In total, eight physicians received no intervention (wait-list control), eight physicians received only the practice-level intervention, eight physicians received only the patient-level intervention, and eight physicians received both the practice - and patient -level intervention. Using the EMR, the practice roster of each participating physician was searched and a list of patients meeting the eligibility criteria and a random number sequence was generated for each stratum separately. Patients were invited to participate according to the random number sequence until the target sample size for each physician was reached or the patient list exhausted. A letter, signed by the physician, was mailed with study documents to invite patients to participate.

The intervention consisted of a practice-level intervention with a Practice Facilitator and a patient-level intervention with a Prevention Practitioner. Training for these roles consisted of: 1) participation in the Clinical Working Group (described below); a two-day training workshop followed by a one-day training workshop before the intervention started; and 3) during the intervention period there was the opportunity to participate in one-hour monthly teleconferences that were facilitated by a member of the Clinical Working Group.

#### Practice-level intervention with a practice facilitator

The multifaceted practice-level intervention was predicated on a model of outreach facilitation [24,25], provided by a trained Practice Facilitator. Each Practice Facilitator supported two Primary Care Team practices (eight physicians). The Practice Facilitator received training in practice facilitation and in the use of the EMR. An EMR audit tool was developed to evaluate each physician’s use of the EMR. Using the EMR audit, potential improvements in the use of the EMR were identified. Each practice-level ‘prescription’ fell into one of five categories: 1) discrete field to check if the a field is available to enter data; 2) data discipline to check if a value is being entered in a consistent location and standard way; 3) registry to determine if a measure can be extracted into a report or patient list; 4) usage to determine if the measure is being used consistently; and 5) resources to assess the functionality of the EMR to facilitate the use of a measure such as reminder or alerts. The Practice Facilitator applied evidence-based strategies including using the EMRs for reminder systems, audit and feedback [28] applying quality improvement techniques, [29] and a needs assessment of barriers and supports to improve CDPS. The Practice Facilitator developed a practice-level ‘prevention prescription’ tailored to the needs of the Primary Care Team practice (see BETTER Trial website for trial-specific tools) [30]. An example could be a prescription to develop a discrete field and data discipline to identify smokers in the practice.

#### Patient-level intervention with a prevention practitioner

Each Primary Care Team practice designated an allied health care professional (e.g., nurse practitioner, nurse or dietician) from within the practice who was trained by the BETTER Trial Clinical Working Group to undertake the role of Prevention Practitioner. Following informed consent, intervention patients were scheduled for a one-hour visit with the Prevention Practitioner. Through motivational interviewing and shared decision-making, a personalized ‘prevention prescription’ was prepared by the Prevention Practitioner during the visit. This prescription was tailored to that patient’s chronic disease risk as determined from their health record and trial-specific “Health Survey” which the patient completed before the visit, which also incorporated their family history (Additional file 1 - Appendix 1). The prevention prescription focused on optimum use of existing capacity, tools and community resources that were available through: 1) the practice itself (e.g., fecal occult blood testing kits, referral to the practice dietician or smoking cessation program); 2) external referrals for tests, specialists, or resources (e.g., screens like mammography, community weight loss programs); and 3) Internet patient resources. CDPS actions were included based on evidence that they lead to clinically important benefits. CDPS actions were identified through a comprehensive review of the literature and approved by the Clinical Working Group (Campbell-Scherer DL, Rogers J, Manca D, et al. Evidence Translation Plan for the BETTER Trial. Manuscript submitted to Can Med Assoc J). (See BETTER Trial website for trial-specific tools [30].)

### Recruitment and follow-up

Patient recruitment occurred August 2010 through April 2011, and follow-up of all patients was completed November 2011. Consenting patients completed the baseline assessment and received the intervention at baseline (T_0_) with outcome assessment at follow-up at 7 months (T_1_).

### Outcome

CDPS actions that have been shown to lead to clinically important benefits for primary prevention of heart disease and diabetes, screening for colorectal, breast, and cervical cancers, and lifestyle factors relevant to chronic disease prevention (alcohol consumption, smoking, diet, and physical activity) were included. Included actions were determined through an evidence review and appraisal by the Clinical Working Group comprised of clinicians with expertise in the relevant clinical areas supported by the Centre for Effective Practice in Toronto, Canada. This yielded a total of 28 actions for which targets were pre-defined (see Additional file 1 - Appendix 2 for a description of the 28 actions, pre-defined targets, and supporting references).

The primary outcome was a composite index, expressed as the ratio (multiplied by 100) of the number of eligible CDPS actions at baseline (denominator) that were subsequently met at follow-up (numerator), measured at the patient level. The composite index was modeled after the Summary Quality Index (SQUID) introduced by Nietert for assessing the quality of primary care interventions [31]. As a function of baseline characteristics, certain individuals were eligible [E] for certain CDPS actions. At follow-up, each patient was re-evaluated and the number of eligible actions which they met [M] were enumerated. For example, eligible actions would be smoking cessation in an actively smoking patient or a mammogram in a patient not up-to-date with her screening mammograms. In this case, the actions would be designated ‘met’ if the patient had quit smoking and had a screening mammogram at follow-up. If the patient had not quite smoking or the mammogram was not completed at follow-up these actions were considered ‘not met’ (Additional file 1 - Appendix 2).

### Measures and data sources

The primary data source was the patient’s EMR. The secondary data source was a trial specific patient-completed Health Survey mailed to each patient at T_0_ and T_1_. The eligible CDPS actions for each patient were determined from the EMR for the two years prior to T_0_ and the baseline Health Survey (Additional file 1 - Appendix 1). The CDPS actions met by each patient were determined from the EMR and the Health Survey at T_1._

### Sample size estimation and statistical analysis

Based on an estimated standard deviation of 20% and an intra-class correlation coefficient of 0.237, we calculated that 896 patients (28 per participating physician) were needed for 80% power to detect an increase in SQUID by 15% or higher with 5% Type I error [32,33].

The analysis was intention to treat. The analytic approach investigated the impact of the intervention (practice-level, patient-level, both practice- and patient-level vs control). The trial design results in patient outcomes being clustered within physician offices. Generalized Estimating Equation (GEE) methods, using a compound symmetric working correlation structure, were used to model this dependence. Generalized score tests were used to assess the significance of potential predictors in this regression framework. Further, Wald tests were used to make comparisons between interventions [34,35]. We used a two-way factorial linear GEE model (i.e., containing a main effect for both the Prevention Practitioner and Prevention Facilitator intervention and an interaction effect between the Prevention Practitioner and the Prevention Facilitator) to assess the impact of each intervention on SQUID. If a non-significant interaction effect was observed we considered a two-way main effects linear GEE model (i.e., containing only a main effect for the Prevention Practitioner intervention and the Prevention Facilitator intervention). The primary analysis involved estimating a two-way factorial linear GEE model adjusting for potential confounders. The statistical data analyses were completed using SAS v.9.3.

### Economic evaluation

Cost-effectiveness analysis from the perspective of the health care payer estimated the cost for improvement in eligible actions met for each of the interventions. Costs included practitioner training and time to administer the intervention, and resources required to accomplish additional actions. The times required to train for and administer the intervention were collected through detailed time logs kept by the Prevention Practitioners and Prevention Facilitators and included in the intervention costs. Time that was specific to conducting the research was also recorded and excluded from the intervention costs (e.g., time spent obtaining patient consent). The resources used were identified from standard costing sources in Ontario and Alberta. (See Additional file 1 - Appendix 3 for list of resources and costs for the economic evaluation.) All costs were in Canadian dollars. The cost-effectiveness estimate was computed as the ratio of the difference between the total costs of each group to the difference in eligible actions met by each group, or incremental cost-effectiveness ratio (ICER). Incremental costs and effects between groups were computed using GEE to account for clustering and adjusted for characteristics presented in Table 1. The results were confirmed using net benefit regression [36].

**Table 1:** Baseline characteristics of patients by randomization group (N = 777)

	**Control**	**PF only**	**PP only**	**PF/PP**
	**(N = 183)**	**(N = 150)**	**(N = 209)**	**(N = 235)**
**Characteristic**				
Age – yr ± SD	54.0 ± 6.4	52.4 ± 7.2	53.3 ± 6.7	52.9 ± 6.9
Female sex -%	69	78	66	77
Minority race or ethnic group -%	11	12	12	11
≥1 yr post-secondary education -%	85	81	88	88
Employment -% Full-time or part-time	70	75	83	74
Marital status -% Married/common-law	73	71	74	83
Total household income -%				
≥ 100,000 CAD	47	47	50	56
≥ 60,000-99,999 CAD	26	28	31	28
Current smoker -%	10	10	15	7
Current alcohol consumption -%				
< 4 times per month	67	65	63	63
≥ 2 times per week	10	10	17	15
Exercise status -%				
Extremely active	20	16	15	21
≤ mildly active	80	84	85	79
Body-mass index^¶^ – mean ± SD	25.2 ± 5.7	25.3 ± 5.1	26.4 ± 5.8	25.0 ± 4.7
Obese -%	14	16	25	16
GAD-7				
Score - mean ± SD	6.0 ± (5.7)	4.8 ± (4.7)	5.7 ± (5.6)	4.8 ± (4.8)
Range	0 to 21	0 to 21	0 to 21	0 to 21
PHQ-9				
Score - mean ± SD	5.4 ± (5.6)	5.2 ± (5.0)	6.2 ± (6.1)	5.0 (± 5.2)
Range	0 to 27	0 to 24	0 to 27	0 to 23
MOS social support score				
– Mean ± SD	74.3 ± (24.6)	73.0 ± (25.9)	75.0 ± (24.7)	79.2 ± (22.1)
Follow-up time – days ± SD	212.3 ± (40.8)	213.9 ± (42.6)	229.6 ± (50.0)	234.6 ± (54.5)

PF: Practice Facilitator (practice-level intervention); PP: Prevention Practitioner (patient-level intervention).

PF/PP: Combined practice-level and patient-level intervention.

CAD: Canadian dollars.

Body mass index (BMI) is the weight in kilograms divided by the square of the height in meters. Obesity is defined as a BMI ≥30.

GAD-7: Generalized Anxiety Disorder. Score for the 7 items ranges from 0 to 21; scores of 5, 10 and 15 represent cut points for mild, moderate and severe anxiety, respectively [37].

PHQ-9: Patient Health Questionnaire-9. Scores for the nine items ranges from 0 to 27. Scores of 5, 15, and 20 represent cut points for mild, moderate and severe major depressive disorder, respectively [38].

MOS Social Support Questionnaire: Scores for the 22 items range from 0 to 100, higher scores indicate higher level of self-perceived support [39].

### Study oversight

Trial oversight, including data management and coordination, was jointly provided by the BETTER team and the Applied Health Research Centre of the Li Ka Shing Knowledge Institute of St. Michael’s Hospital, Toronto, Canada. The trial was approved by the Ontario Cancer Research Ethics Board (REB), University of Alberta REB, and all relevant REBs in each province and at each Primary Care Team site.

## Results

### Patients

**Figure 2:**
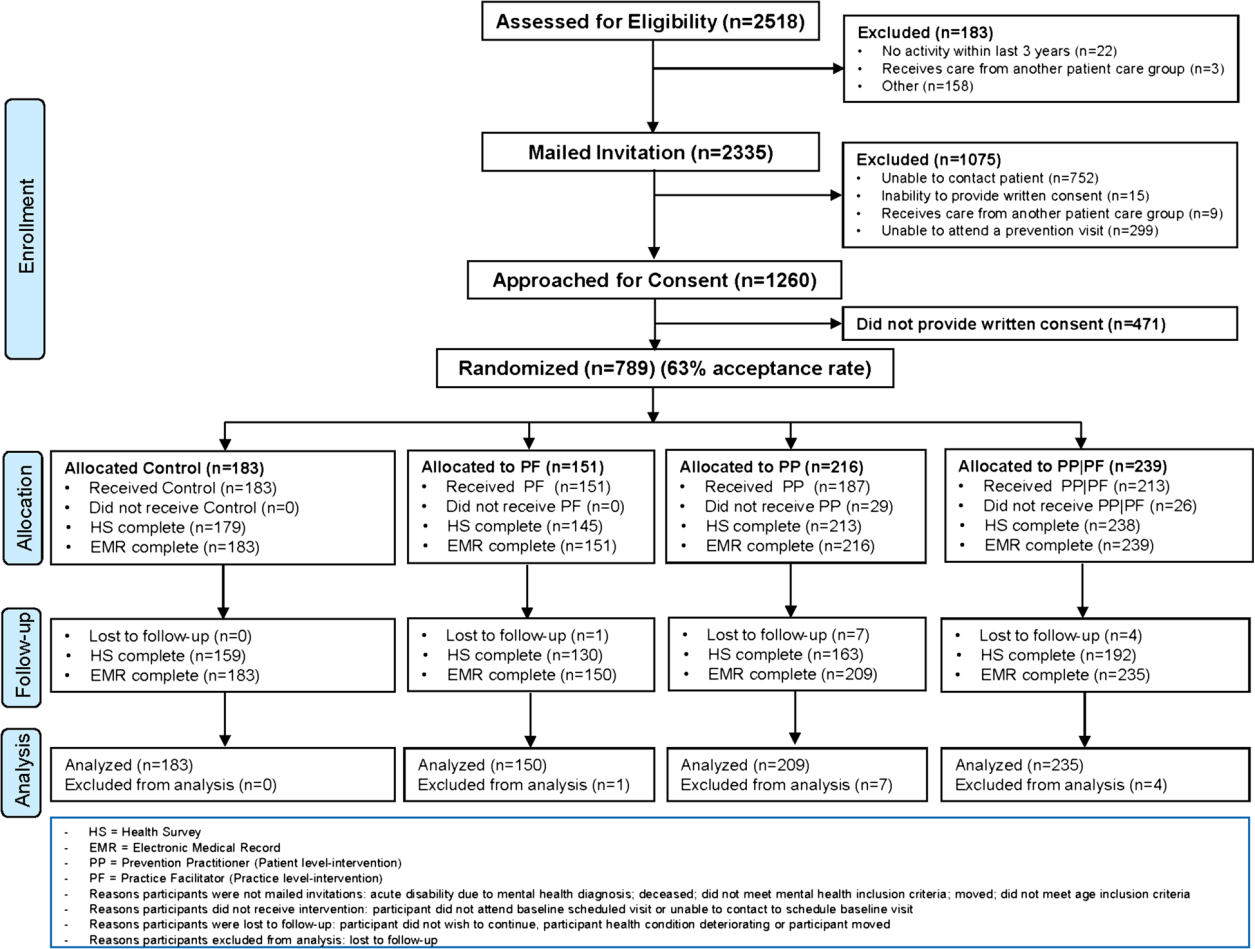
Enrollment, randomization, and follow-up of patients.

There were 789 patients enrolled in the trial among 1,260 patients approached for consent, representing an acceptance rate of 63%. Of these, 12 withdrew consent, leaving 777 for analysis. As shown in the CONSORT diagram (Figure 2), the return rate at T_0_ was 98.2% and similar across all four randomizations groups; the return rate at T_1_ was 81.6% ranging from 75.5% to 86.9% across groups. Baseline characteristics were balanced across groups (Table 1). Baseline eligibility for each of the 28 CDPS actions is presented in Table 2. The mean number of CDPS actions for which patients were eligible at baseline was 8.96 (SD 3.20).

**Table 2:** Baseline eligibility of patients for prevention and screening actions by randomization group N (%)

	**Control**		**PF only**		**PP only**		**PF/PP**	
**Prevention and screening actions (N=, eligible)**								
1. Fasting blood sugar screening	61	(33.3)	35	(23.3)	77	(36.8)	82	(34.9)
2. Fasting blood sugar monitoring	8	(4.4)	4	(2.7)	13	(6.2)	3	(1.3)
3. Blood pressure screening	107	(58.5)	82	(54.7)	117	(56.0)	136	(57.9)
4. Blood pressure monitoring	40	(21.9)	44	(29.3)	50	(23.9)	58	(24.7)
5. Hypertension treatment	23	(12.6)	23	(15.3)	20	(9.6)	26	(11.1)
6. Framingham calculated	108	(59.0)	68	(45.3)	114	(54.5)	132	(56.2)
7. Framingham improved	21	(11.5)	15	(10.0)	25	(12.0)	19	(8.1)
8. LDL improved	22	(12.0)	18	(12.0)	20	(9.6)	21	(8.9)
9. Cholesterol treatment	22	(12.0)	19	(12.7)	20	(9.6)	21	(8.9)
10.Breast cancer screening (women only; N = 561)	48	(38.1)	40	(34.2)	51	(37.0)	59	(32.8)
11. Colorectal cancer screening	61	(33.3)	41	(27.3)	68	(32.5)	56	(23.8)
12. Cervical cancer screening (women only; N = 561)	43	(34.1)	35	(29.9)	37	(26.8)	51	(28.3)
13. BMI screening	43	(23.5)	22	(14.7)	58	(27.8)	41	(17.4)
14. Waist circumference measured	173	(94.5)	141	(94.0)	177	(84.7)	223	(94.9)
15. Weight control	94	(51.4)	87	(58.0)	129	(61.7)	134	(57.0)
16. Weight control referral	94	(51.4)	87	(58.0)	130	(62.2)	135	(57.4)
17. Smoking screening	31	(16.9)	30	(20.0)	18	(8.6)	85	(36.2)
18. Smoking cessation	22	(12.0)	18	(12.0)	36	(17.2)	22	(9.4)
19. Smoking cessation referral	22	(12.0)	18	(12.0)	36	(17.2)	22	(9.4)
20. Alcohol screening	61	(33.3)	36	(24.0)	36	(17.2)	96	(40.9)
21. Alcohol control	28	(15.3)	32	(21.3)	42	(20.1)	49	(20.9)
22. Alcohol cessation referral	28	(15.3)	32	(21.3)	42	(20.1)	49	(20.9)
23. Physical activity screening	162	(88.5)	138	(92.0)	164	(78.5)	222	(94.5)
24. Physical activity ≥ 90 minutes/week	91	(49.7)	65	(43.3)	109	(52.2)	125	(53.2)
25. Physical activity program referral	91	(49.7)	65	(43.3)	109	(52.2)	125	(53.2)
26. Nutrition screening	125	(68.3)	66	(44.0)	129	(61.7)	139	(59.1)
27. Healthy diet score improved	15	(8.2)	10	(6.7)	20	(9.6)	13	(5.5)
28. Nutrition counseling referral	15	(8.2)	10	(6.7)	20	(9.6)	13	(5.5)

PF: Practice Facilitator (practice-level intervention).

PP: Prevention Practitioner (patient-level intervention).

PF/PP: Combined practice-level and patient-level intervention.

Body mass index (BMI) is the weight in kilograms divided by the square of the height in meters. Obesity is defined as a BMI ≥30.

### Primary outcome

As shown in Table 3, the number of eligible CDPS actions for each patient was balanced across groups. Considering all patients, the mean SQUID was significantly higher for patients receiving the Prevention Practitioner intervention compared to controls, and compared to those receiving the Prevention Facilitator intervention. Considering unadjusted means, control patients met 21.0% of target actions, compared to 28.4% in the Prevention Facilitator only group, 53.6% in the Prevention Practitioner only group, and 58.4% in the Prevention Facilitator/Prevention Practitioner group (Wald test; PP versus control, P < 0.001) (Table 3).

**Table 3:** Prevention and screening actions by randomization group and strata (Mean ± SD)

	**Control**	**PF only**	**PP only**	**PF/PP**	P value*
**All patients; N**	183	150	209	235	
Eligible actions	9.1 ± 3.4	8.5 ± 3.2	8.9 ± 3.2	9.2 ± 3.1	0.57
Actions met	1.9 ± 1.8	2.6 ± 2.3	4.7 ± 2.7	5.3 ± 2.6	<0.001
SQUID	21.0 ± 17.5	28.4 ± 23.6	53.6 ± 26.0	58.4 ± 23.8	<0.001
Adjusted SQUID	23.1 (19.2 – 27.1)	28.5 (20.9 – 36.0)	55.6 (49.0 – 62.1)	58.9 (54.7 – 63.1)	
**Stratum 1:**					
**General health patients: N**	119	107	129	158	
Eligible actions	8.9 ± 3.3	8.4 ± 3.1	8.5 ± 3.0	9.0 ± 3.0	0.80
Actions met	1.9 ± 1.7	2.7 ± 2.3	4.8 ± 2.5	5.3 ± 2.5	<0.001
SQUID	21.5 ± 16.8	30.3 ± 24.1	57.7 ± 25.1	59.7 ± 23.4	<0.001
Adjusted SQUID	23.5 (19.3 – 27.7)	31.6 (22.9 – 40.4)	60.0 (52.8 – 67.2)	60.3 (55.6 , 65.1)	
**Stratum 2:**					
**Mental health patients; N**	64	43	80	77	
Eligible actions	9.5 ± 3.5	8.8 ± 3.4	9.6 ± 3.3	9.6 ± 3.5	0.56
Actions met	1.9 ± 1.8	2.4 ± 2.2	4.5 ± 2.9	5.3 ± 2.9	<0.001
SQUID	20.1 ± 18.7	23.6 ± 21.7	47.1 ± 26.2	55.7 ± 24.8	<0.001
Adjusted SQUID	21.0 (13.3 – 28.8)	21.3 (11.9 – 30.6)	46.6 (39.4 – 53.7)	57.0 (52.3 – 61.7)	

PF: Practice Facilitator (practice-level intervention).

PP: Prevention Practitioner (patient-level intervention).

PF/PP: Combined practice-level and patient-level intervention.

SQUID: Summary Quality Index defined is the ratio of the number of CDPS actions met according to pre-defined targets to the number of actions for which the patient was eligible.

*P values are based on two-sided Generalized Score Tests for equality of means across 4 groups.

The unadjusted two-way factorial linear GEE model suggested that there was no significant interaction effect (P = 0.654). The two-way main effects linear GEE model showed that the effect of Prevention Practitioner intervention was significant (P < 0.001) while the Prevention Facilitator was not (P = 0.085). The estimated ICC was 0.381 in the overall sample.

After adjusting for potential confounders, in the two-way factorial linear GEE analysis, patients in the control group met 23.1% (95% CI: 19.2% to 27.1%) of actions, compared to 28.5% (95% CI: 20.9% to 36.0%) in the Prevention Facilitator only group, 55.6% (95% CI: 49.0% to 62.1%) in the Prevention Practitioner only group, and 58.9% (95% CI: 54.7% to 63.1%) in the PF/PP group. The impact of the Prevention Practitioner intervention was significant (P < 0.001) while the impact of the Prevention Facilitator intervention remained not significant (P = 0.16) (Additional file 1 - Appendix 4). No other covariates had a statistically significant impact.

In the stratum of the general health patients, the adjusted difference in SQUID between the Prevention Facilitator/Prevention Practitioner group and the control group was 36.8%; whereas, in the mental health stratum the adjusted difference in SQUID between the Prevention Facilitator/Prevention Practitioner group and control group was 36.0%. This difference was not statistically significant (P = 0.68). None of the other differences-in-difference estimates were statistically significant.

**Figure 3:**
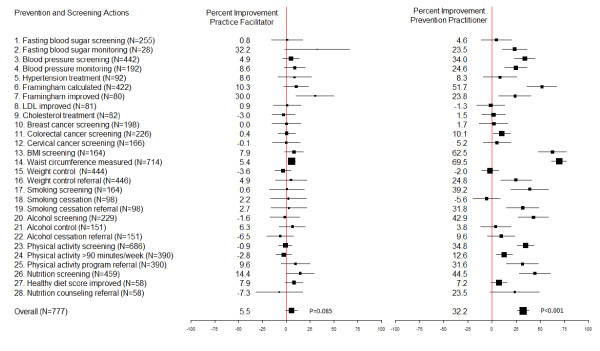
**Improvement in prevention and screening actions for practice facilitator and prevention practitioner groups compared to controls.** Legend: FBS = Fasting Blood Sugar; BP = Blood Pressure; LDL = Low-density Lipoprotein; CRC = Colorectal Cancer; BMI: Body Mass Index.

The effect of the Prevention Facilitator and Prevention Practitioner interventions for each of the 28 components of the SQUID are presented graphically in Figure 3. These estimates are displayed for descriptive purposes only since the trial was not powered to study each CDPS action separately.

### Economic evaluation

**Figure 4:**
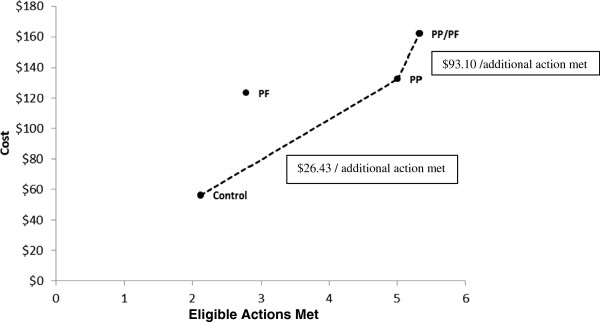
**Costs and effects for control and treatment groups.** The ratio of the difference in costs between two groups to the difference in eligible actions accomplished represents the incremental cost-effectiveness ratio (ICER) (dotted line). PP/PF: Combined practice-level and patient-level intervention. NB: The PF intervention is within the “efficiency frontier” so it is not considered an efficient use of resources.

The Prevention Practitioner intervention cost an additional $76.21 per patient compared to control. The ICER was $26.43 per additional action met for the patient-level intervention compared to control (95% CI $16 to $44). The Prevention Facilitator intervention had both higher costs and lower effectiveness relative to the combination of Prevention Practitioner and control groups, suggesting it was not efficient (Figure 4). The Prevention Facilitator/Prevention Practitioner intervention cost an additional $29.53 over the PP alone, resulting in an ICER of $93.10 per additional action accomplished (95% CI not defined [36]).

## Discussion

This trial showed that a Prevention Practitioner, taken from within the practice and trained to conduct dedicated prevention visits at which each patient was given a tailored prevention prescription and directed to relevant practice or community resources, improved CDPS by 32.5% compared to control. The benefit of the Prevention Practitioner intervention was seen in both general health and mental health strata.

A meta-analysis conducted by Baskerville and colleagues showed an overall effect size of 0.56 favoring practice facilitation. Applying Baskerville’s approach calculating effect size to this study resulted in an effect size of 1.45 for the patient-level intervention and an effect size of 0.36 for the practice-level intervention. In the Baskerville meta-analysis, tailoring and intensity of practice-level interventions were two attributes that had a significant impact on the size of the effect [24]. It may be that these attributes were not optimally accomplished in the implementation of the practice-level intervention of this trial. Another possible explanation for why we did not find a benefit for the practice-level intervention is that the seven-month timeframe was too short to detect an effect for the intervention that only indirectly affected patients. Conversely, the patient-level intervention with the Prevention Practitioner had a direct impact on patient outcomes.

Our aim was to develop an intervention that was feasible and practical to implement in the primary care setting, and pragmatic in its design to enhance generalizability and relevance [40,41]. The Prevention Practitioner intervention was a one hour visit to conduct evidence-based shared decision making around CDPS with the patient. The product was a personalized prescription where the patient was given relevant tools and referral to relevant resources. This co-ordination visit was designed to make it feasible to implement in the primary care setting. It is recognized that changing lifestyle factors generally requires more intensive interventions than provided in this trial. Nevertheless, assessment and advice in primary care can effectively help patients modify their behaviour [42,43]. The trial was powered to detect a difference in SQUID and, accordingly, the cost-effectiveness analysis considered the extra cost of an improved CDPS related to SQUID. Since multiple chronic diseases are positively impacted by the intervention, the added cost of the Prevention Practitioner intervention is likely small in comparison to the long-term costs of managing these chronic diseases. In addition, we did not capture costs savings associated with avoiding unnecessary actions, which if included could make the intervention more attractive. We feel this represents good value for money; however, decision makers must consider how much they are willing to pay to increase CDPS actions.

The principal limitation of the study is that it involved urban Primary Care Team practices that had adopted EMRs and, therefore, may not be representative of other settings (e.g., solo or rural practices). However, the findings are relevant to most countries as the use of EMRs and shift to team-based ‘medical homes’ as the locus of primary care, are the direction that primary care is developing in many countries [44]. The intervention was multifaceted, where an amalgam of evidence-based strategies was applied. The design of the study does not allow for distinguishing the effect of each individual strategy separately. The language and literacy requirements of informed consent and completion of the Health Survey would impact participating patients and, therefore, could limit generalizability to patients without those skills [45]. Although this trial was not designed or powered to test the effect of the intervention on each action separately, with respect to lifestyle factors the effect was not detected for smoking cessation (-5.6%) and weight control (-2%), while there was an improvement in physical activity (12.6%) and healthy diet (7.2%) compared to controls. It is possible that a larger effect might be achieved with greater intensity or longer follow-up. Of the 28 actions in the SQUID, 5 were considered ‘met’ if they were recorded in the EMR (Additional file 1: Appendix 2, Items 6, 14, 17, 29, 23). We included these actions because the evidence review highlighted the importance of recoding this information in the EMR to improve CDPS practices. However, these 5 actions were balanced across randomization groups (Table 2) and would, therefore, have similar weighting across all groups.

## Conclusions

This trial provides strong support for the benefits of a multifaceted facilitation intervention directed at clinically important CDPS actions tailored to the individual patient. The intervention was also effective for patients with moderate mental illness. The cost-effectiveness of analysis suggests that the patient-level intervention with a Prevention Practitioner visit is economically attractive, particularly when considering the potential of preventing chronic diseases [1,46]. Although measuring long-term outcomes was beyond the scope of this trial, the included CDPS actions were specifically selected because the evidence base was already strong that they lead to improved long-term outcomes.

The BETTER Trial developed a framework that bridged the gap of chronic disease prevention and screening knowledge to practice through an intervention that integrated evidence-based actions for patients 40 to 65 years of age, which were adapted to the practice setting. The patient-level intervention with a Prevention Practitioner is a modification of practice facilitation, This modification of the role involves an allied health professional from within the team practice, specially trained in evidence-based chronic disease prevention and screening actions, skills, tools and resources applied through direct patient contact at a dedicated ‘prevention’ visit.

The role of a Prevention Practitioner who, through training, develops skills and expertise in CDPS can be a resource to a specific practice or shared among several practices. The BETTER Trial team continues to update training resources and tools [30], and are currently testing adaptations of the Prevention Practitioner intervention in different settings across Canada, including those with large rural and remote populations.

## Abbreviations

BETTER Trial: Building on existing tools to improve chronic disease prevention and screening in primary care; CDPS: Chronic disease prevention and screening; EMR: Electronic medical record; GEE: Generalized estimating equation; ICER: Incremental cost-effectiveness ratio; PF: Practice facilitator (implemented the practice-level intervention); PP: Prevention practitioner (implemented the patient-level intervention); RCT: Randomized controlled trial; REB: Research ethics board; SQUID: Summary quality index.

## Competing interests

The authors declare that they have no competing interests.

## Authors’ contributions

The BETTER Trial Investigators are: *Writing Committee* – EG (PI), DM (co-PI), JR, MM*; Methodology Working Group*: EG (Chair), DM, RM, KT, MM, PK; *Statistical Analysis*: RM, KT, CM; *Trial Coordination* – Applied Health Research Centre, LP, CA*; Economic Analysis* – JH, JB; *Clinical Working Group* – DCS (Chair), RB, SB, SB, SB, JC, MD, SD, MG, CH, LH, MK, DK, CK, KLR, JM, VM, JP, LR, JR, GS, LS, RS, DLV, RW, MY. All authors read and approved the final manuscript.

## Pre-publication history

The pre-publication history for this paper can be accessed here:

http://www.biomedcentral.com/1471-2296/14/175/prepub

## Supplementary Material

Additional file 1 Supplementary appendices. Click here for file
