# Supplementary material for: Improving chronic disease prevention and screening in primary care: results of the BETTER pragmatic cluster randomized controlled trial
Source: BMC Fam Pract. 2013 Nov 20;14:175. doi: 10.1186/1471-2296-14-175 (PMC4225577; doi:10.1186/1471-2296-14-175)
Supplement: Additional file 1 — Supplementary appendices. [file 1471-2296-14-175-S1.doc]

**Supplementary Appendices**

Table of Contents

Appendix 1: Components and Sources of Patient-completed Health Survey

Appendix 2: Chronic Disease Prevention and Screening Actions and Targets

Appendix 3: Resources and Costs for Economic Evaluation

Appendix 4: Adjusted Linear GEE Analysis

**Appendix 1: Components and Sources of Patient-completed Health Survey**

The **BETTER Health Survey** contains 88 items (many of which include one or more sub questions) and 11 sections (see below). Space is provided for participants at the end of the survey to capture comments.

The ***General Information*** section includes 7 single-item questions about gender, age, height, weight, and blood pressure (3 questions). These questions are taken or modified from the Canadian Community Health Survey (CCHS) [1] or Ontario Health Survey (OHS) [3].

The ***Screening Tests*** section includes 9 single-item questions for both men and women related to colorectal cancer screening and an additional 9 single-item questions for women related to breast cancer screening. These questions are taken or modified from the OHS [3]

The ***Chronic Health Conditions*** section includes 12 questions for both men and women about whether or not they currently have asthma/emphysema/chronic bronchitis, arthritis/rheumatism, diabetes (with 2 sub questions), digestive problems, heart trouble, HIV illness or AIDS, kidney disease, liver problems, stroke, and cancer (3 questions for men and women and 2 additional questions for women). These questions include a modified version of the self-report Charlson Comorbidity Index [2] which has a standard scoring for this scale, single-item diabetes questions from the nurse interview component of the OHS [3] and the CCHS, and 4 single-item questions that have been developed specifically for the BETTER project (whether the patient has ever been diagnosed with breast, colorectal, ovarian or cervical cancer).

The ***Lifestyle*** section includes 16 single-item questions related to smoking (4 questions), alcohol consumption (6 questions), and exercise (6 questions). These questions are taken or modified from the CCHS, Rollnick’s Readiness Ruler [4], a single-item from the Stanford Patient Education Centre’s “Self-efficacy for Managing Chronic Disease 6-item scale”[5], and a single-item global health assessment of physical activity question [6]

The ***General Health*** section includes 10 questions (with numerous sub questions) related to measuring general health, anxiety, and depression. The complete 12-item SF-12v2 [7], 7-item GAD-7 [8], and 9-item PHQ-9 [9] will also be included in order to assess general health, anxiety and depression respectively. Standard scoring procedures will be used for each of these 3 scales.

The ***Support Systems*** section includes 2 questions, 1 question about the number of close friends and relatives and 1 question (with numerous sub questions) about how often support was available in a variety of situations. These questions are taken from the CCHS, based on the MOS Social Support Survey [10] that allows for the calculation of an overall score and the following 4 subscales scores: tangible social support; affection; positive social interaction; and emotional or informational support.

The ***Questions About Health Services*** section includes 3 single-item questions about access to, quality of, and satisfaction with health care services received in the past 6 months. These questions are taken or modified from the CCHS.

The ***Medications*** section includes 2 questions, 1 single-item question about the number of prescription medications being taken and 1 question about whether the patient is taking medications for blood pressure, diabetes, cholesterol, smoking cessation, alcohol cessation or other reasons. The first question is from the nurse interview component of the OHS, while the second question has been developed specifically for the BETTER project.

The **About You** section includes 9 single-item demographic questions including citizenship (2 questions), ethno-cultural background, education level, employment status, marital status, household income, and subjective social status (2 questions). These questions are taken or modified from the CCHS, Canadian Census [11], OHS, and the MacArthur Scale of Subjective Social Status (both the SES ladder and community ladder) [12].

The ***Family Medical History*** section includes 2 questions, 1 single-item question about ethnic/cultural background and 1 comprehensive question designed to collect information about the illness history (diabetes, heart disease, breast cancer, colorectal cancer, ovarian cancer) of various family members (mother, father, sisters, brothers, daughters, sons, and grandparents/aunts/uncles/nieces/nephews on the mother’s and father’s side). These questions have been developed specifically for the BETTER project and are derived loosely from the “My Family Health Portrait” tool [13, 14] and minimum family history data recommended in the literature[15, 16]

The ***Dietary Assessment*** section includes 4 questions (with numerous sub questions) related to food avoidance (5 questions), preparedness to improve diet, confidence about improving diet, and a detailed weekly food consumption question that collects information about frequency of consumption and serving size for a comprehensive list of meats, eggs, dairy, cheese, frozen desserts, fried foods, baked goods, convenience foods, table fats, and snacks. The food avoidance questions are from the CCHS and will combine into a standard scale score; the preparedness to improve diet is a single-item that will be adapted from Rollnick’s Readiness Ruler; the single-item confidence question will be adapted from the Stanford Patient Education Centre’s “Self-efficacy for Managing Chronic Disease 6-item scale”; and the food consumption question will produce a standard score based on the MEDIFICTS dietary questionnaire [17, 18].

The **BETTER Follow-up Health Survey** is identical to the BETTER Health Survey but excludes the following sections and questions that do not change from one survey to the next:

- Gender, age and height were excluded from the ***General Information*** section
- The ***Support Systems*** section
- The ***About You*** section
- The ***Family Medical History*** section

**References for Appendix 1**

1. Canadian Community Health Survey (CCHS) - Cycle 1.1. Ottawa: Statistics Canada. (Accessed August 14, 2012, at http://www.statcan.gc.ca/concepts/health-sante/index-eng.htm.)

2. Chaudhry S, Jin L, Meltzer D. Use of a self-report-generated Charlson Comorbidity Index for predicting mortality. Med Care 2005;43(6):607-15.

3. The Ontario Health Survey: 1996/97 & 1990. Toronto: Association of Public Health Epidemiologists in Ontario. (Accessed August 14, 2012, at http://www.apheo.ca/index.php?pid=211#96OHS.)

4. Rollnick S. Health Behaviour Change: A Guide for Practioners. 8th Revised edition ed: A Churchill Livingston Title; 1999.

5. Lorig KR, Sobel DS, Ritter PL, Laurent D, Hobbs M. Effect of a self-management program on patients with chronic disease. Eff Clin Pract 2001;4(6):256-62.

6. Blair SN, Haskell WL, Ho P, et al. Assessment of habitual physical activity by a seven-day recall in a community survey and controlled experiments. Am J Epidemiol 1985;122(5):794-804.

7. Ware J, Jr., Kosinski M, Keller SD. A 12-Item Short-Form Health Survey: construction of scales and preliminary tests of reliability and validity. Med Care 1996;34(3):220-33.

8. Spitzer RL, Kroenke K, Williams JW, Löwe B. A brief measure for assessing generalized anxiety disorder: The gad-7. Arch Intern Med 2006;166(10):1092-7.

9. Kroenke K, Spitzer RL, Williams JB. The PHQ-9: validity of a brief depression severity measure. J Gen Intern Med 2001;16(9):606-13.

10. Sherbourne CD, Stewart AL. The MOS social support survey. Soc Sci Med 1991;32(6):705-14.

11. Census 2006 - 2B (Long Form). Statistics Canada, 2006. (Accessed January 24, 2012, at http://www23.statcan.gc.ca/imdb-bmdi/pub/instrument/3901_Q2_V3-eng.pdf.)

12. The MacArthur Scale of Subjective Social Status. San Francisco: University of California, San Francisco, 2007. (Accessed June 23, 2008, at http://www.macses.ucsf.edu/research/psychosocial/subjective.php.)

13. My Family Health Portrait: A tool from the Surgeon General. Maryland: National Institutes of Health, 2009. (Accessed August 14, 2012, at https://familyhistory.hhs.gov/fhh-web/home.action.)

14. Facio FM, Feero WG, Linn A, Oden N, Manickam K, Biesecker LG. Validation of My Family Health Portrait for six common heritable conditions. Genet Med;12(6):370-5.

15. Qureshi N, Carroll JC, Wilson B, et al. The current state of cancer family history collection tools in primary care: a systematic review. Genet Med 2009;11(7):495-506.

16. Wilson BJ, Qureshi N, Santaguida P, et al. Systematic review: family history in risk assessment for common diseases. Ann Intern Med 2009;151(12):878-85.

17. Kris-Etherton P, Eissenstat B, Jaax S, et al. Validation for MEDFICTS, a dietary assessment instrument for evaluating adherence to total and saturated fat recommendations of the National Cholesterol Education Program Step 1 and Step 2 diets. J Am Diet Assoc 2001;101(1):81-6.

18. Taylor A, Wong H, Wish K, et al. Validation of the MEDFICTS dietary questionnaire: A clinical tool to assess adherence to American Heart Association dietary fat intake guidelines. Nutrition Journal 2003;2(1):4.

**Appendix 2: Chronic Disease Prevention and Screening Actions and Targets**

1. **Summary Quality Index (SQUID) TABLE** which defines the expected (E) referral/action based on baseline assessment and what the target M is at follow-up.

| **ITEM #** | **Item (E)** | **Eligibility Criteria (E) = Baseline** | **Target (M) at 6 Months Since Baseline** | **References** |
| --- | --- | --- | --- | --- |
| 1 | FBS Screen | All non-diabetics with at least one risk factor (see Section B) that haven’t had an Fasting Blood Sugar (FBS) in the past year | FBS completed | 1, 2, 3, 19 |
|  |  | All non-diabetics without risk factors (see Section B) that haven’t had an FBS in the past 3 years | FBS completed | 2, 19 |
| 2 | FBS Monitor | All non-diabetics with impaired FBS (6 - 6.9) | FBS completed AND referral to health professional or specialist complete for either nutrition/diet or diabetes | 1, 2, 3 |
|  |  |  | FBS completed AND referral to a program complete for any one of nutrition/diet; physical activity/exercise or weight control | 1, 2, 3, 7, 9 |
|  |  |  | FBS completed AND discussion with health professional occurred for any one of nutrition/diet; physical activity/exercise; diabetes or weight control | 1, 2, 3, 9 |
| 3 | BP Screen | All non CVD patients AND Non-hypertensive and Non-diabetic > 12 months since BP check | BP checked | 1, 4, 5 |
|  |  | All non CVD patients AND Non-hypertensive and Diabetics > 6 months since BP check | BP checked | 2, 5 |
|  |  | All non CVD patients AND Hypertensive patients > 6 months since BP check | BP checked | 4, 5 |
| 4 | BP Monitor | All non CVD patients with hypertension | BP checked since baseline AND non-diabetic with BP < 140/90 | 4, 5 |
|  |  | All non CVD patients with hypertension | BP checked since baseline AND diabetic with BP <130/80 | 1, 4 |
| 5 | Hypertension Treatment | All non CVD patients with hypertension AND Non-diabetic with BP ≥ 140/90 OR Diabetic with BP ≥ 130/80 | Non-diabetic with BP < 140/90 OR Diabetic with BP <130/80 OR; | 4 |
|  |  |  | Newly prescribed hypertension medication OR | 1, 4 |
|  |  |  | 1 of the following 3 options:   - Referral to health professional or specialist complete for either nutrition/diet or Hypertension OR - Referral to a program complete for any one of: Visit to hypertension clinic; Nutrition/diet; Physical activity/exercise OR - Discussion with health professional occurred for any one of: Nutrition/diet; Physical activity/exercise; Hypertension or Hypertension medication | 4, 3, 7 |
| 6 | Framingham Measure | All non-diabetic Men ≥ 40, Women ≥ 50 without CVD with Framingham never calculated | Framingham calculated in EMR since baseline | 4, 5 |
|  |  | All non-diabetic Men ≥ 40, Women ≥ 50 without CVD with Framingham screening for any one of the following:   - Framingham last calculated > 3 years ago and 10 year risk is < 10% - Framingham last calculated > 1 year ago and 10 year risk is 10% - 20% - Framingham 10 year risk is > 20% | Framingham calculated in EMR since baseline | 4, 5 |
| 7 | Framingham Improve | All non-diabetics Men > 40, Women > 50 without CVD, with LDL ≥ 3.5 for Framingham moderate & high risk patients (score ≥ 10) | Framingham calculated value decreased or no increase since baseline*** | 4, 5 |
|  |  | All non-diabetics Men > 40, Women > 50 without CVD, with LDL ≥ 5 for Framingham low risk patients (score <10) | Framingham calculated value decreased or no increase since baseline*** | 4, 5 |
| 8 | LDL Improve | All non-CVD patients not currently on a statins and Non-diabetic with LDL ≥ 3.5 for Framingham moderate & high risk patients (score >10) | LDL < 3.5 | 4, 5 |
|  |  | All non-CVD patients not currently on a statins and Non-diabetic LDL ≥ 5 for Framingham low risk patients (score < 10) | LDL < 5 | 4, 5 |
|  |  | All non-CVD patients not currently on a statins and Diabetic with LDL ≥ 2 | LDL < 2 | 4, 5 |
| 9 | Cholesterol Treatment | All non-CVD patients not currently on a statins and Non-diabetic with LDL ≥ 3.5 for Framingham moderate & high risk patients (score >10) | Prescribed cholesterol medication (e.g., statin) OR 1 of the following:   - Referral to health professional or specialist complete for Nutrition/diet - Discussion with health professional occurred for any one of Nutrition/diet; Physical activity/exercise or Cholesterol medication | 3, 4, 5, 7 |
|  |  | All non-CVD patients not currently on a statins and Non-diabetic LDL ≥ 5 for Framingham low risk patients (score < 10) | Prescribed cholesterol medication (e.g., statin) OR 1 of the following:   - Referral to health professional or specialist complete for Nutrition/diet - Discussion with health professional occurred for any one of Nutrition/diet; Physical activity/exercise or Cholesterol medication | 3, 4, 5, 7 |
|  |  | All non-CVD patients not currently on a statins and Diabetic with LDL ≥ 2 |  |  |
| 10 | Breast Cancer Screen | All women ≥ 50-65 without a personal history of breast cancer AND without family history risk of breast or ovarian cancer (see Section B):   - - Routine mammogram not done within 2 years OR | Mammography complete since baseline | 6, 8, 20 |
|  |  | All women 40-65 without personal history of breast cancer but with a family history risk of breast or ovarian cancer (see Section B) ≤50 years of age:   - - Routine mammogram not done within 1 year | Mammography complete since baseline | 15, 16, 17, 18, 20 |
| 11 | CRC Screen | All patients ≥50-65 without personal history CRC and without family history of CRC (see Section B) with any one of the following:   - - FOBT not done within 2 years (Ontario) OR FOBT not done within 1 year (Alberta)   - Sigmoidoscopy not done within 5 years   - Colonoscopy not done within 10 years if normal OR Colonoscopy not done within 3 years if abnormal | CRC screening completed since baseline | 7, 8, 11, 22 |
|  |  | All patients 40-65 with a family history of CRC (see Section B) with any one of the following:   - - FOBT not done within 2 years (Ontario) OR FOBT not done within 1 year (Alberta)   - Sigmoidoscopy not done within 5 years   - Colonoscopy not done within 10 years if normal OR Colonoscopy not done within 3 years if abnormal | CRC screening completed since baseline | 8, 13, 15, 16, 17, 18, 22 |
| 12 | Cervical Cancer Screen | All women without personal history of cervical cancer with no pap tests within the past 3 years OR | Pap test complete since baseline | 8, 10, 14 |
|  |  | All women without personal history of cervical cancer with any abnormal pap tests within the past 3 years |  |  |
| 13 | BMI Screen | BMI not recorded in EMR | BMI captured in the EMR | 2, 4, 7 |
|  |  | Weight not recorded in EMR | Weight captured in the EMR | 2, 7 |
| 14 | Waist Circumference | Waist circumference not captured in EMR | Waist Circumference captured in EMR | 4, 5, 7 |
| 15 | Weight Control | BMI ≥ 25 | Decrease in BMI or no increase in BMI | 2, 4 |
|  |  |  | Decrease in weight or No increase in weight |  |
| 16 | Referral Weight Control | BMI ≥ 25 | BMI < 25 OR | 2, 3, 4, 7 |
|  |  |  | Referral to health professional or specialist complete for nutrition/diet OR | 2, 3, 4, 7 |
|  |  |  | Referral to program complete for any one of Nutrition/diet; Physical activity/exercise; Weight control program OR | 2, 3, 4, 7 |
|  |  |  | Discussion with health professional occurred for any one of Nutrition/diet; Physical activity/exercise; Weight control | 2, 3, 4, 7 |
|  |  | High waist circumference (see Section C) | Normal Waist Circumference (see SECTION C) OR | 4, 5 |
|  |  |  | Referral to health professional or specialist complete for nutrition/diet OR | 2, 4, 5, 7 |
|  |  |  | Referral to program complete for any one of Nutrition/diet; Physical activity/exercise; Weight control program OR | 2, 4, 5, 7 |
|  |  |  | Discussion with health professional occurred for any one of Nutrition/diet; Physical activity/exercise; Weight control | 2, 4, 5, 7 |
| 17 | Smoking Screen | Smoking status not recorded in EMR | Smoking status captured in EMR | 2, 3, 4, 7, 12 |
| 18 | Smoking Cessation | Smoker | Stopped smoking | 2, 3, 4, 7, 12 |
| 19 | Referral Smoking Cessation | Smoker | Newly prescribed smoking cessation medication | 2, 3, 4, 7, 12 |
|  |  |  | Referral to smoking cessation program made or complete | 3, 4, 7, |
|  |  |  | Discussion to health professional regarding smoking cessation or smoking cessation medication occurred | 2, 3, 4, 7 |
| 20 | Alcohol Screen | Alcohol consumption not recorded in EMR | Alcohol consumption recorded in EMR | 3, 4, 7 |
|  |  | Patient at risk drinker status not recorded in EMR | Is patient is an at-risk drinker is captured in EMR | 3, 4, 7 |
| 21 | Alcohol Control | ≥ 9 drinks per week for Women | < 9 drinks per week for Women | 4, 7, 19 |
|  |  | ≥ 14 drinks per week for Men | < 14 drinks per week for Men |  |
| 22 | Referral Alcohol Cessation | ≥ 14 drinks per week for Men | Newly prescribed alcohol cessation medication OR | 19 |
|  |  |  | Referral for at risk drinking counseling or program made or complete, OR | 19 |
|  |  |  | Discussion with health professional regarding alcohol cessation medication or alcohol cessation occurred | 4, 19 |
|  |  | ≥ 9 drinks per week for Women* | Newly prescribed alcohol cessation medication OR | 19 |
|  |  |  | Referral for at risk drinking counseling or program made or complete, OR | 19 |
|  |  |  | Discussion with health professional regarding alcohol cessation medication or alcohol cessation occurred | 4, 19 |
| 23 | Physical Activity Screen | Details regarding physical activity are not recorded in the EMR | Details regarding patient physical activity data captured in the EMR since baseline | 2, 4 |
| 24 | Physical Activity 90min per week | <90 minutes exercise/week** | Patient exercising ≥90 minutes per week since baseline | 2, 4 |
| 25 | Referral Physical Activity Program | <90 minutes vigorous exercise/week** | Referral for physical activity/exercise program made or complete | 2, 4 |
|  |  |  | Discussion with health professional regarding physical activity/exercise occurred | 2 |
| 26 | Nutrition Screen | Diet/nutrition details not recorded in EMR | Diet/nutrition details captured in the EMR | 2, 4 |
| 27 | Healthy Diet Score | Diet score that indicates an unhealthy diet (≥70) | Healthy diet score (<70) | 21 |
| 28 | Referral Nutrition Counseling | Diet score that indicates an unhealthy diet (≥70) | Referral for nutrition counseling/dietitian or nutrition program made or complete | 2, 4 |
|  |  |  | Discussion with health professional regarding nutrition/diet occurred | 2, 4 |

* note the revised guideline is ≥7.

** note the minimum target is 150 minutes moderate intensity, the CWG felt that change behavior for very sedentary individuals would be an initial target of 90 minutes.

*** In order to not double count improvement in BP & cholesterol, this parameter for Framingham chosen. The goal was to capture worsening in status.

1. Indicators and Ranges

| **Domain** | **Target/Range** | **References** |
| --- | --- | --- |
| FBS | Normal <6mmol/L; Impaired Fasting Glucose 6 – 6.9 mmol/L; Suspected Diabetes/Diabetes >6.9 mmol/L | 1, 2 |
| BP – No hypertension, no CVD | Patients without diabetes target is ≤140/90 (recheck 12 months) and Patients with diabetes target is ≤ 130/80 (recheck 6 and 12 months) | 4, 5 |
| BP – With hypertension, no CVD | Patients without diabetes target is ≤140/90 (> 140/90 recheck 6 and 12 months) and Patients with diabetes target is ≤ 130/80 (> 130/80 recheck 6 and 12 months) | 1 |
| Diabetes | if not risk factors order fasting blood sugar (FBS) every 3 years or if risk factors order FBS every year | 1, 2, 3 |
| Diabetes Risk Factors | Risk Factors: one or more from below patient at risk | 1 |
|  | 1^st^ degree relative with Diabetes | 1 |
|  | Ethnic (Aboriginal, African, Asian, Hispanic) | 1 |
|  | Impaired fasting blood glucose (6.0-6.9) or impaired glucose tolerance in the past year (up to 3 values) | 1 |
|  | Hypertension or increase blood pressure or on medications for hypertension | 1, 3 |
|  | History of gestational diabetes | 1 |
|  | Obese (BMI ≥30) | 1 |
|  | High waist circumference | 1 |
| Framingham score | For non-diabetics Men ≥ 40 and Women ≥ 50 | 4, 5 |
|  | - Low risk : 10 year CVD related death or non-fatal MI risk less than 10% and Calculate Framingham every 3 years | 5 |
|  | - Moderate risk : 10 year risk of 10% to 20% and Calculate Framingham every year | 5 |
|  | - High risk : 10 year risk over 20% and Calculate Framingham at 12 months | 5, 18 |
| Nutrition/Diet | Target:≥70 = need dietary changes; 40 – 69 = heart healthy diet; <40 = Therapeutic Lifestyle Change (TLC) diet  *(score <40 points indicates intake of <7% of energy from saturated fat, <30% of energy from total fat, and <200 mg dietary cholesterol/day).* See Medficts tool for calculations | 2, 4, 18, 23 |
| Physical Activity | Target = ≥ 90 minutes of moderate activity per week | 2, 4 |
| Alcohol | At Risk Drinker: ≥ 2 standard drinks on any one day OR ≥ 9 drinks for women/week, ≥14 drinks men/week | 4, 19 |
| BMI | Value: Underweight: <18.5; Normal:18.5 – 24.9; Overweight: 25-29.9; Obese: ≥30  Follow-up BMI Calculation: take #1 from follow-up survey and #3 from baseline survey  Calculation: kg/m² (weight in kilograms divided by height in meters squared) | 2, 4 |
| Waist Circumference | Value: Normal: < 102 cm for males or < 90 cm for S. Asian, Japanese, Chinese, ethic south and central American and First Nations men and < 88 cm for females; High: Males ≥ 102cm (40 inches) or ≥ 90 cm for S. Asian, Japanese, Chinese, ethic south and central American and First Nations men Females ≥ 88 cm (35 inches) | 1, 4, 5 |
| Breast Cancer: Family Risk Factors | Any 1st or 2nd degree relative female or male with any breast or ovarian cancer on either side of the family | 15, 16, 17, 18, 20 |
| Breast Cancer Test and Review Frequency | All women ≥ 50 without family history risk of Breast or Ovarian Cancer - Routine mammogram every 2 years | 6, 8, 20 |
|  | All women 40-65 with a family history risk of Breast or Ovarian Cancer – Routine mammogram done every year | 20 |
| Family History Risk of colorectal Cancer (CRC) | Low Risk: 1st degree relative with CRC > 35 years of age; or 2nd degree relatives with CRC 35-50 years of age; or ≥ two 2nd degree relative with CRC > 50 years of age. Moderate Risk: 1st or 2nd degree relative with CRC ≤ 35 years of age | 15, 16, 17, 18, 22 |
| Testing and Review Frequency | FOBT every 2 years (Ontario)/ every 1 years (Alberta); -Sigmoidoscopy every 5 years; -Colonoscopy every 10 years if normal; if abnormal every 3 years | 7, 8 , 11 |
| Cervical Cancer PAP Test and Review Frequency | Ontario – if negative in 3 consecutive years and no pap done in previous 3 years; Alberta – if negative in 3 over 5 years and no pap done in previous 3 years; General - < 3 test in past 5 years with no abnormalities | 10,14 |

Legend: FBS = Fasting Blood Sugar; BP = Blood Pressure; CVD = Cardiovascular Disease; MI = Myocardial Infarction; BMI = Body Mass Index; LDL = Low-density Lipoprotein; CRC = Colorectal Cancer; EMR = Electronic Medical Record; FOBT = Fecal Occult Blood Test; PAP = Papanicolaou

1. **References for Appendix 2**
2. 2008 Clinical Practice Guidelines. Toronto, ON: Canadian Diabetes Association, 2008. (Accessed August 14, 2012, at http://www.diabetes.ca/for-professionals/resources/2008-cpg/.)
3. Clinical practice recommendations 2009. American Diabetes Association, 2010. (Accessed August 14, 2012, at http://care.diabetesjournals.org/content/33/Supplement_1.)
4. Screening for Type 2 Diabetes Mellitus in Adults, Topic Page. Rockville, MD: U.S. Preventive Services Task Force, 2008. (Accessed August 14, 2012, at http://www.uspreventiveservicestaskforce.org/uspstf/uspsdiab.htm.)
5. Risk Estimation and the Prevention of Cardiovascular Disease. Edinburgh, UK: Scottish Intercollegiate Guidelines Network, 2008. (Accessed August 14, 2012, at http://www.sign.ac.uk/guidelines/fulltext/97/index.html.)
6. Genest J, McPherson R, Frohlich J, et al. 2009 Canadian Cardiovascular Society/Canadian guidelines for the diagnosis and treatment of dyslipidemia and prevention of cardiovascular disease in the adult - 2009 recommendations. Can J Cardiol 2009;25(10):567-79.
7. Screening for breast cancer: U.S. Preventive Services Task Force recommendation statement. Ann Intern Med 2009;151(10):716-26.
8. The guide to clinical preventive services 2009: Recommendations of the United States Preventive Services Task Force. Rockville, MD: U.S. Preventive Services Task Force. (Accessed August 14, 2012, at http://graphics8.nytimes.com/packages/pdf/health/09PATIENT_1.pdf.)
9. Health Care Guideline: Preventive Services for Adults. Bloomington, MN, 2011. (Accessed August 14, 2012, at http://www.icsi.org/preventive_services_for_adults/preventive_services_for_adults_4.html.)
10. Suspected Cancer in Primary Care: Guidelines for investigation, referral and reducing ethnic disparties. Wellington, NZ: New Zealand Guidelines Group; 2009.
11. Cervical Screening: Practice Guideline Report. Toronto, Ontario: Cancer Care Ontario, 2005. (Accessed August 15, 2012, at http://web.archive.org/web/20060623065117/http://www.cancercare.on.ca/pdf/pebc_cervical_screen_s.pdf.)
12. Screening for colorectal cancer: U.S. Preventive Services Task Force recommendation statement. Ann Intern Med 2008;149(9):627-37.
13. Diagnosis and Management of Lung Cancer Executive Summary: ACCP Evidence-Based Clinical Practice Guidelines Northbrook, IL: American College of Chest Physicians, 2007. (Accessed August 15, 2012, at http://journal.publications.chestnet.org/issue.aspx?journalid=99&issueid=22060&direction=P.)
14. Clinical Practice Guideline: Screening for Colorectal Cancer. Edmonton, AB: Toward Optimized Practice Program, 2008. (Accessed August 15, 2014, at http://www.topalbertadoctors.org/download/304/colorectal_guideline.pdf.)
15. Guideline For Screening For Cervical Cancer. Edmonton, AB: Toward Optimized Practice, 2011. (Accessed August 15, 2012, at http://www.topalbertadoctors.org/download/587/cervical%2Bcancer%2Bguideline.pdf.)
16. Wilson BJ, Qureshi N, Santaguida P, et al. Systematic review: family history in risk assessment for common diseases. Ann Intern Med 2009;151(12):878-85.
17. Family History and Improving Health. Rockville (MD): Agency for Healthcare Research and Quality (US), 2009. (Accessed August 15, 2012, at http://www.ncbi.nlm.nih.gov/books/NBK32554/.)
18. Plat AW, Kroon AA, Van Schayck CP, De Leeuw PW, Stoffers HE. Obtaining the family history for common, multifactorial diseases by family physicians. A descriptive systematic review. Eur J Gen Pract 2009;15(4):231-42.
19. Clinical Utility of Cancer Family History Collection in Primary Care. Rockville, MD: Agency for Healthcare Research and Quality (US), 2009. (Accessed at http://www.ncbi.nlm.nih.gov/books/NBK38760/.)
20. A Pocket Guide for Alcohol Screening and Brief Intervention. Rockville, MD: National Institute on Alcohol Abuse and Alcoholism, 2005. (Accessed August 14, 2012, at http://pubs.niaaa.nih.gov/publications/practitioner/pocketguide/pocket.pdf.)
21. Hereditary Breast Cancer (HBC) Risk Triage. Toronto, ON: Canadian Cancer Society, 2001. (Accessed August 15, 2012, at http://www.mountsinai.on.ca/care/family-medicine-genetics-program/resources/HBC%20triage%20card_1335932073.pdf.)
22. National Cholesterol Education Program. Second Report of the Expert Panel on Detection, Evaluation, and Treatment of High Blood Cholesterol in Adults (Adult Treatment Panel II). Circulation 1994;89(3):1333-445.
23. Colorectal Cancer (CRC) Risk Management Recommendations. Toronto, ON: Canadian Cancer Society, 2004. (Accessed August 15, 2012, at http://www.mountsinai.on.ca/care/family-medicine-genetics-program/resources/CRC%20Triage%20card_2760642125.pdf.)
24. National Cholesterol Education Program. Second Report of the Expert Panel on Detection, Evaluation, and Treatment of High Blood Cholesterol in Adults (Adult Treatment Panel II). Circulation 1994;89(3):1333-445

**Appendix 3: Resources and Costs for Economic Evaluation**

| **Item type** | **Examples of SQUID items** | **Resource use** | **Cost and source (ONT)**  **Canadian Dollars** |
| --- | --- | --- | --- |
| **Laboratory screening** | -FBS screening or monitoring  -Framingham or LDL improvement | Laboratory tests  PCP visit (control)* | $10.34 (FBS)^1^  $22.23 (Total and HDL cholesterol, lipids)^1^ |
| **Measurement or recording of data in EMR** | -BP screening or monitoring  -Framingham measurement  -BMI, waist circumference, or  weight control monitoring  -Alcohol or smoking cessation screening or monitoring  -Physical activity or nutrition screening or monitoring | 5 mins nursing (control)† | $36.85 (RN hourly wage)^2^ |
| **Screening procedures** | Breast cancer screening  -CRC screening  -Cervical cancer screening | Procedures  Incentives (ON)  PCP visit (control)* | $83.67 (Mammogram)^2, 3^  $21.31 (Occult blood, incentives)^1, 3^  $360.00 (Sigmoidoscopy)^4^  $440.00 (Colonscopy)^4, 5^  $11.55 (Pap smear add-on fee)^3^  $13.96 (Cervical vaginal specimen)^1^  $6.86 (Incentive fees)^3^ |
| **Risk factor treatment** | Treatment of hypertension or high cholesterol | PCP visit, or other recorded referral (PP patients)  Medications not included‡ | $34.70 (Intermediate assessment)^3^ |
| **Referrals to allied health professionals** | -Smoking cessation  -Alcohol cessation  -Nutrition counseling | Counseling (1- 6 one hour sessions) with nurse, pharmacist, or dietician | $36.85 (RN hourly wage)^2^  $41.81 (pharmacist hourly)^2^  $34.34 (dietician hourly)^2^ |
| **Referrals to community-based programs** | -Weight control  -Smoking cessation  -Alcohol cessation  -Physical activity/exercise | Out-of-pocket costs (not included) | N/A |
| **Primary Care physician (PCP) visits*** | N/A | Intermediate or general assessment | $34.70 (Intermediate)^3^  $77.20 (General)^3^ |

**SQUID items categorized by types of resources used and the unit costs associated with the achievement of each item. Ontario costs shown.**

*Unless otherwise indicated, we assumed one PCP visit occurred for each control patient. When only one screening test was ordered, we assumed this occurred during an PCP visit billed as an intermediate assessment, and when multiple screening tests were ordered, we assumed this occurred during a comprehensive assessment or general physical exam.

†For items that only required a small measurement or recording of data in EMR, we assumed that this was achieved with 5 minutes of nursing time, so as to not overestimate the cost of such items with a separate visit cost. Such items are achieved through the course of the prevention visit for PP intervention patients.

‡Medications are not funded by the Ministry of Health and Long Term Care for the patient population in the BETTER study.

**Appendix 4: Adjusted Linear GEE Analysis**

|  | Beta  (95% CI) | P Value* |
| --- | --- | --- |
| PP | 0.32  (0.25 to 0.40) | <0.001 |
| PF | 0.05  (-0.03 to 0.14) | 0.160 |
| PP/PF | -0.02  (-0.14 to 0.10) | 0.739 |
| Age (continuous) | -0.001  (0.00 to 0.00) | 0.295 |
| Female Gender | -0.02  (-0.06 to 0.03) | 0.449 |
| Not Caucasian Ethnicity | 0.04  (-0.03 to 0.11) | 0.320 |
| Any Postsecondary Education | -0.03  (-0.09 to 0.04) | 0.442 |
| Full/Part-time Employment | 0.01  (-0.04 to 0.05) | 0.772 |
| Married or Common Law | -0.02  (-0.06 to 0.02) | 0.242 |
| Income 60,000-99,999 CAD | 0.04  (-0.01 to 0.09) | 0.100 |
| Income > 100,000 CAD | 0.03  (-0.03 to 0.09) | 0.311 |
| Smokers | -0.04  (-0.08 to 0.004) | 0.111 |
| Alcohol 2-4x per month | 0.002  (-0.05 to 0.05 | 0.926 |
| Alcohol >= 2x per week | 0.04  (-0.02 to 0.10) | 0.216 |
| Extremely Active | -0.04  (-0.07 to 0.001) | 0.070 |
| BMI (continuous) | -0.001  (-0.006 to 0.003) | 0.595 |
| Obese (BMI > 30) | -0.01  (-0.08 to 0.05) | 0.662 |
| PHQ-9 (continuous) | 0.0001  (-0.005 to 0.005) | 0.956 |
| GAD-7 (continuous) | -0.003  (-0.01 to 0.003) | 0.309 |
| MOS Social Support Score (continuous) | 0.004  (-0.002 to 0.00) | 0.220 |
| Mental Health | -0.05  (-0.09 to -0.001) | 0.047 |
| Ontario | 0.03  (-0.03 to 0.09) | 0.310 |

* P values are based on two-sided using Wald tests.
